# Primary Hepatic Mucosa-Associated B-Cell Lymphoma in a Patient with Primary Sclerosing Cholangitis—A Case Ultimately Requiring Liver Transplantation

**DOI:** 10.3390/diagnostics15162082

**Published:** 2025-08-19

**Authors:** Jerica Novak, Mihajlo Đokić, Miha Petrič, Diana Vozlič, Milanka Živanović, Branislava Ranković, Blaž Trotovšek

**Affiliations:** 1 Department of Abdominal Surgery, University Medical Center Ljubljana, Zaloska 7, 1000 Ljubljana, Slovenia; mihajlo.dokic@kclj.si (M.Đ.); miha.petric@kclj.si (M.P.); blaz.trotovsek@kclj.si (B.T.); 2 Medical Faculty Ljubljana, University of Ljubljana, Vrazov trg 2, 1000 Ljubljana, Slovenia; 3 Clinical Institute of Radiology, University Medical Center Ljubljana, Zaloska 7, 1000 Ljubljana, Slovenia; diana.vozlic@kclj.si; 4 Institute for Pathology, Faculty of Medicine, University in Ljubljana, Korytkova 2, 1000 Ljubljana, Slovenia; milanka.zivanovic@mf.uni-lj.si (M.Ž.); branislava.rankovic@mf.uni-lj.si (B.R.)

**Keywords:** primary MALT lymphoma, liver, primary sclerosing cholangitis, small-for-size syndrome, liver transplantation

## Abstract

**Background:** Primary hepatic extranodal marginal zone lymphoma of mucosa-associated type (MALT) is an extremely rare liver neoplasm. The lesions are often misdiagnosed for the most common primary hepatic malignancy, such as hepatocellular carcinoma and cholangiocarcinoma. As the diagnosis is most often made after the resection, there are still no clear guidelines for the optimal treatment of these patients. **Case Presentation:** A 30-year-old male patient with known primary sclerosing cholangitis (PSC) was treated at the Department of Abdominal Surgery Ljubljana due to a mass in the right liver, believed to be an intrahepatic cholangiocarcinoma. Due to the extent of the disease, extended right hepatectomy with the resection of the hepatocholedochus, lymphadenectomy, and hepaticojejunal anastomosis were performed. After the surgery, the patient developed a small-for-size syndrome and therefore necessitated a liver transplantation (LT) that was afterwards successfully performed. **Discussion:** This case highlights the diagnostic challenges of differentiating primary hepatic MALT lymphoma from cholangiocarcinoma on imaging, especially in patients with underlying liver disease. Preoperative confirmation of the malignant disease could potentially change treatment course in our patient. Therefore, a serious surgical complication with development of small-for-size syndrome after major hepatectomy could potentially be prevented. Regarding the underlying liver disease, the patient could probably be a candidate for LT with the bridging chemotherapy. **Conclusions:** Primary hepatic MALT lymphoma is an extremely rare liver lesion but remains a valid option in a differential diagnosis of liver lesions in patients with chronic viral infection or autoimmune disease, especially in settings of cirrhosis. Moreover, a high level of suspicion must be raised in young patients with solitary liver mass and autoimmune liver disease. Surgical resection is the best way to achieve elimination of the disease.

## 1. Background

Primary hepatic extranodal marginal zone lymphoma of mucosa-associated type (MALT) is an extremely rare liver neoplasm [1]. As the liver lesions in lymphoma formation are nonspecific not only in clinical and laboratory findings but also in imaging characteristics, definitive preoperative diagnosis of primary liver MALT lymphoma is extremely challenging. They are often misidentified for the most common primary hepatic malignancy, such as hepatocellular carcinoma and cholangiocarcinoma [2]. Primary liver MALT lymphoma represents a rare subtype of primary hepatic lymphoma (PHL). Due to its scarcity, there are no clear guidelines regarding the diagnostic workup, imaging, treatment, and follow-up [3,4,5]. Definitive diagnosis is based on pathology findings [6]. According to the literature review, there are only 53 papers with reported primary MALT lymphoma [3]. Nevertheless, it has been shown that surgery is a suitable treatment modality, with or without additional treatments [3,4,5,6,7,8,9,10,11,12,13,14,15,16,17,18,19,20,21], in treatment of primary hepatic MALT lymphoma.

In this article, we present a rare case of primary hepatic MALT lymphoma mimicking mass forming intrahepatic cholangiocarcinoma in a young male patient with primary sclerosing cholangitis (PSC). Due to the extent of the disease, extended right hepatectomy with the resection of the hepatocholedochus, lymphadenectomy, and hepaticojejunal anastomosis were performed After the initial surgery, he developed small-for-size syndrome and therefore necessitated a liver transplantation (LT) that was afterwards successfully performed.

## 2. Case Presentation

### 2.1. History and Laboratory Findings

A 30-year-old male with known PSC was treated at the Department of the Abdominal Surgery at the University Medical Centre Ljubljana due to a solitary mass in the right liver lobe. The patient was regularly followed at the gastroenterology outpatient clinic. In addition to the PSC, he also had psoriasis with no other known comorbidities. During the follow-up, seven years after the first outpatient clinic visit, a tumour formation in the right liver lobe was firstly described on abdominal MR imaging. The patient was otherwise asymptomatic with laboratory results as follows: WBC 4.1 × 10^9^/L, platelet 170 × 10^9^/L, HB 160 g/L, AST 0.69 μkat/L, ALT 1.21 μkat/L, gamaGT 1.6 μkat/L, alkaline phosphatase 2.28 μkat/L, bilirubin total 10 μmol/L, bilirubin direct 3 μmol/L, INR 0.97, LDH 2.61 μkat/L, CK 1.61 μkat/L, alpha fetoprotein 3.8 kU/L, Ca 19-9 < 2 kU/L, and CEA 4.4 μg/L.

### 2.2. Imaging

**Figure 1:**
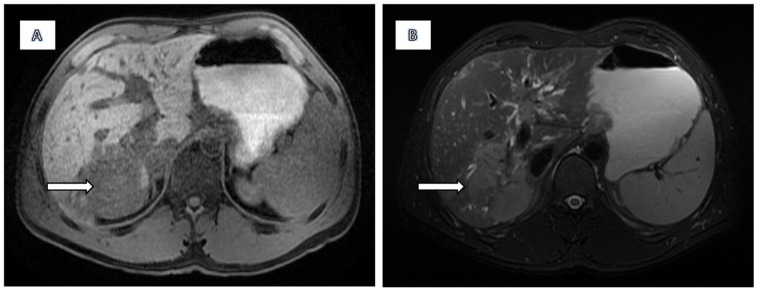
The lesion is moderately hypointense on T1-weighted images (**A**) and moderately hyperintense on T2 fat-suppressed sequences (**B**).

**Figure 2:**
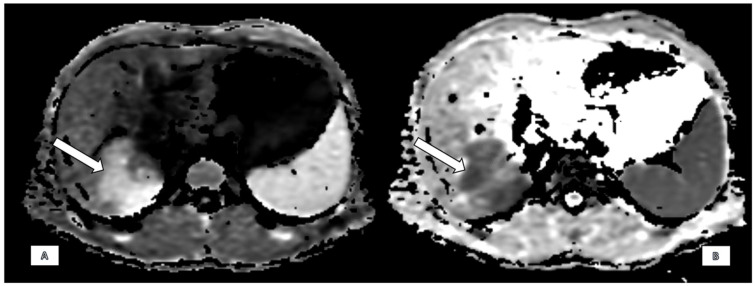
Diffusion-Weighted Imaging (DWI): There is marked diffusion restriction with low apparent diffusion coefficient (ADC) values (**A**,**B**).

**Figure 3:**
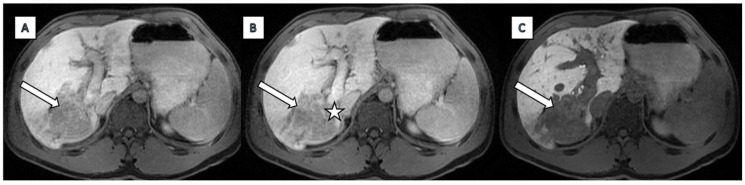
Dynamic Contrast Enhancement: The lesion remains mostly hypointense throughout the arterial (**A**), portal venous (**B**), delayed, and hepatobiliary (**C**) phases. This lack of arterial phase hyperenhancement and washout is atypical for hepatocellular carcinoma and more consistent with hypovascular tumours such as cholangiocarcinoma or lymphoma. Vascular Involvement (star): There is occlusion of the posterior right portal vein extending to the right portal vein bifurcation, and the right hepatic vein is also occluded. These findings suggest an infiltrative aggressive process.

Findings on MRCP with hepatospecific liver contrast demonstrated a solitary, ill-defined focal mass centred in the right hepatic lobe, predominantly involving segments 7, 8, and 6 and measuring approximately 72 × 62 mm. The lesion was moderately hypointense on T1-weighted images and hyperintense on T2 fat-saturated sequences (Figure 1). Diffusion-weighted imaging revealed marked diffusion restriction within the lesion (Figure 2). There was no evidence of signal drop on in-phase and out-of-phase sequences, effectively excluding intracellular fat content. Following contrast administration, subtle enhancement was seen, but mostly the lesion remained hypointense across all dynamic phases, including the hepatobiliary phase (Figure 3). Vascular assessment showed occlusion of the right portal vein extending up to portal vein bifurcation. The right hepatic vein was not visualised and presumed occluded. Close contact with vena cava inferior was detected. Although there was no clear imaging evidence of direct adrenal gland invasion, the close proximity of the mass and the presence of indistinct fat planes raised suspicion for extrahepatic spread. No satellite lesions were identified in the left hepatic lobe. There was marked atrophy of the right hepatic lobe, likely secondary to portal vein occlusion, with compensatory hypertrophy of the left hepatic lobe.

**Figure 4:**
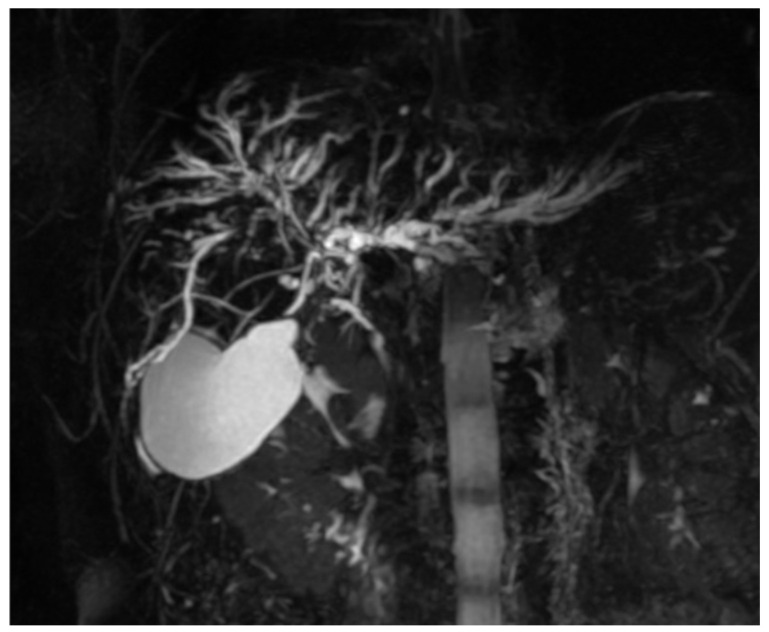
Biliary Tree: MRCP demonstrates multifocal annular short segment strictures alternating with areas of normal and mildly dilated intrahepatic bile ducts producing the classic beaded appearance of PSC. These changes are more pronounced in the right hepatic lobe, where the mass is located, reflecting combined effects of PSC and tumour-related biliary involvement.

Additional findings included multifocal, short segmental, annular strictures of the intrahepatic bile ducts with alternating normal and mildly dilated segments, producing a characteristic “beaded” appearance on MRCP (Figure 4). These findings were consistent with PSC, with right lobe changes compounded by the adjacent mass. The left lobe demonstrated PSC-related strictures predominantly in the lateral segments. Splenomegaly with the spleen measuring 15 cm was noted. A small likely hamartomatous lesion was noted. No free fluid or enlarged lymph nodes were identified in the upper abdomen. Regarding the tumour characteristics with the large infiltrative-appearing mass in the right hepatic lobe with vascular involvement and suspected extrahepatic spread, the mass was suspicious for intrahepatic cholangiocarcinoma in the background of PSC.

### 2.3. Primary Surgery

Patient documentation was discussed at the multi-disciplinary team meeting. Due to the suspected intrahepatic cholangiocarcinoma in the background of PSC exploration with hepatic resection was indicated without prior liver biopsy. Liver biopsy was not performed due to strong suspicion of cholangiocarcinoma in a patient with underlying PSC with radiologically extensive disease, as biopsy would further delay the surgery. Due to the extent of the disease, extended right hepatectomy with the resection of the hepatocholedochus, lymphadenectomy, and hepaticojejunal anastomosis were performed. During the surgery, frozen sections of lymph nodes along the common hepatic artery and resection margins of the hepaticus were negative; the specimen from the tumour itself was not taken for frozen section, as the tumour was believed to be cholangiocarcinoma.

### 2.4. Pathology Findings

**Figure 5:**
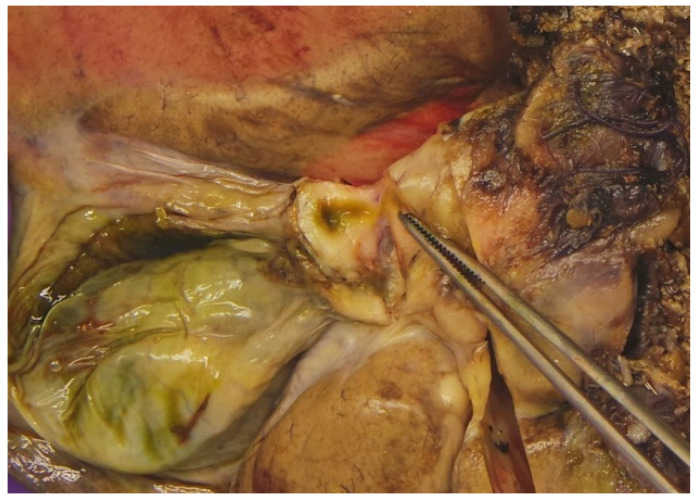
Gross findings on pathomorphology with the thickened cystic duct.

On cross-section, a 9 × 7 cm large tumour mass was present alongside the course of bile duct. The tumour mass was light yellowish pink on the cut surface and had the appearance of fish flesh. The liver parenchyma outside the described tumour was macroscopically unremarkable. Numerous bile ducts, along with the cystic duct, were thickened, with narrowed lumen (Figure 5). Representative samples have been taken for pathohistological evaluation.

**Figure 6:**
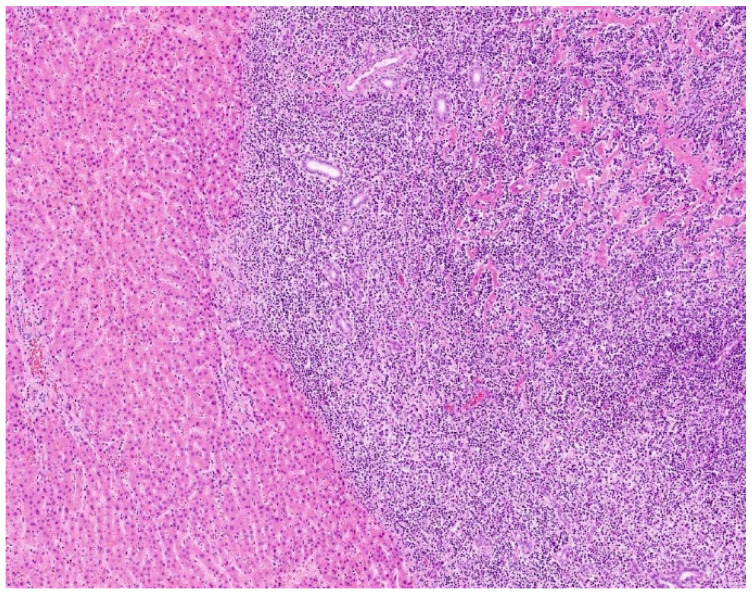
Histopathology—Liver parenchyma infiltrated with dense lymphoid infiltrate.

On histology, tumour was composed of multifocal nodular atypical lymphoid proliferation with multiple reactive lymphoid cells in liver samples and in the cystic duct. Lymphoid follicles were composed predominantly of small- to medium-sized lymphocytes, with a focal presence of larger lymphocytes (Figure 6).

**Figure 7:**
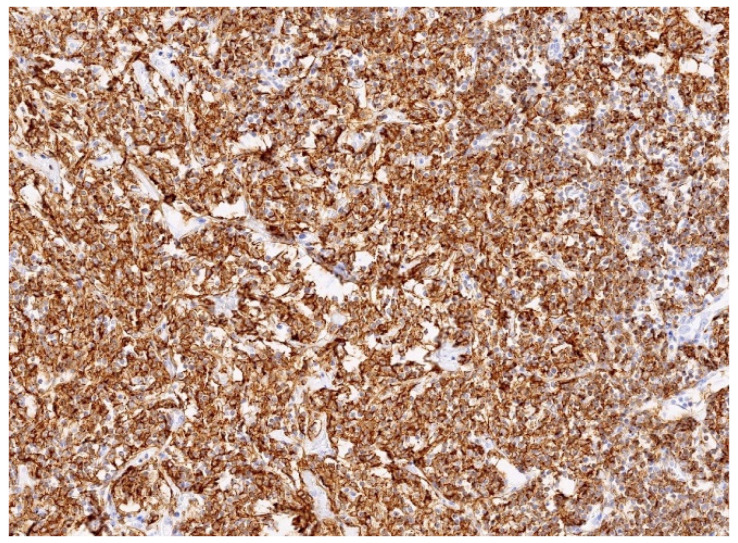
Immunohistochemical staining with CD20 showed predominance of B-cells.

Immunophenotyping showed a significant predominance of B lymphocytes (CD20) over T lymphocytes (CD3) within the infiltrate. B lymphocytes were negative for CD5, cyclin D1, CD10, and BCL6 (Figure 7). The proliferation index Ki67 was approximately 15%. CD23 and Bcl6 showed focal colonisation of pre-existing lymphoid follicles by small B lymphocytes. Molecular genetic analyses were performed to determine the clonality of B lymphocytes and showed monoclonal B lymphocytes with rearrangements in the immunoglobulin heavy chain (IGH-FR1 and IGH-FR2 regions) and light chain kappa (IGK). Morphology and additional immunohistochemical as well as molecular genetic analyses were consistent with the diagnosis of MALT lymphoma.

### 2.5. Postoperative Course, Liver Transplantation, and Follow-Up

**Figure 8:**
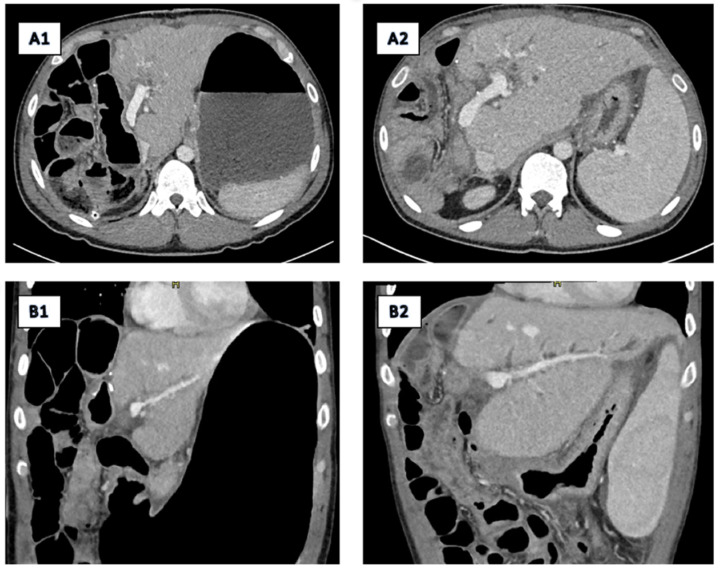
Postoperative CT scans obtained immediately after extended right hepatectomy (**A1**,**B1**) and at six-month follow-up (**A2**,**B2**) demonstrate hypertrophy of the liver remnant. Despite this compensatory regeneration, the residual liver volume remained insufficient to meet metabolic demands. The patient developed liver failure progressing to multiorgan dysfunction, consistent with small-for-size syndrome—a serious postoperative complication arising from an inadequate functional liver remnant following major hepatic resection.

On the fourth postoperative day (POD), liver failure with kidney failure began to develop. On the seventh POD, the patient developed multiorgan failure requiring vasoactive support. The leading factor for multiorgan failure was liver failure due to the small liver remnant that resulted in transient encephalopathy, ascites, and hyperbilirubinemia. With conservative measures, liver function was stabilised, but the patient remained icteric. On the follow-up CT of the abdomen, no necrosis of the liver remnant was described, vessels were patient with no prominent thrombosis (Figure 8(A1,B1)). A fine needle biopsy of the third liver segment was performed and revealed cholestatis hepatitis with acute cholangitis. A diagnosis of small-for-size syndrome was established. A CT scan obtained at six-month follow-up (Figure 8(A2,B2)) demonstrated hypertrophy of the liver remnant. Despite this compensatory regeneration, the residual liver volume remained insufficient to meet metabolic demands.

Staging of the lymphoma, including FDG-PET, was performed and revealed that the disease was limited to the liver. The hematooncologic department was consulted, and as the disease was resected, the patient was disease-free with favourable prognosis and low risk of disease progression. No additional treatment was planned. The patient became a candidate for LT due to small-for-size syndrome with worsening function of the liver remnant. The patient was therefore placed on the Eurotransplant list for LT. The patient was discharged from the surgical ward with stable liver function but present hyperbilirubinemia with consequent icterus. Later on, the patient was hospitalised at the gastroenterology ward due to worsening of the liver function with febrile state and infection of unknown origin.

Four months after the initial surgery, LT was successfully performed. Histology of the liver remnant revealed acute suppurative cholangitis with portal fibrosis with no remains of lymphoma. On the 14th post-transplantation day, elevation of the liver enzymes was noted, and liver biopsy was performed. The histology revealed an acute cellular liver rejection with the rejection activity index at 5, but moreover, there was also centrilobular necrosis of 20% of hepatocytes possibly due to the reperfusion injury. The CMV staining was negative. The patient received higher dose steroid treatment, and the liver test with liver function normalised. The patient was successfully discharged on the 25th post-transplantation day.

The patient is under active surveillance at the gastroenterology outpatient clinic, now 48 months after the LT. Follow-up is complex and consists of screening regarding the possible lymphoma recurrence, follow-up of the liver function and PSC, and screening for possible inflammatory bowel disease. Currently he is receiving immunosuppressive therapy with no additional therapy regarding MALT lymphoma. He remains lymphoma-free with stable liver function.

## 3. Discussion

We present a rare case of primary hepatic MALT lymphoma in a young male patient with underlying PSC and an unusual course of surgical treatment ending in a successful LT.

PHL represents only a small number of all liver malignancies. Most of the cases are predominantly of B-cell origin. The ethology of PHL occurrence is still unclear, but association with infection and compromised immunity have been reported as possible factors [7]. Marginal zone lymphomas (MZLs) are a group of clinically indolent B-cell lymphomas postulated to derive from memory B lymphocytes in the ‘marginal zone’ of secondary lymphoid tissue. The MZL term encompasses distinct entities with shared phenotypic and genotypic features. These entities include extranodal marginal zone B-cell lymphoma (EMZL), MALT lymphoma, splenic MZL, and nodal MZL [22,23]. MALT lymphoma is most commonly found in stomach, skin, lung, head, and neck regions. However, occurrence in liver is rare [23].

Regardless of the location, MALT lymphomas are composed of heterogeneous small B-cells, including marginal zone (centrocyte-like) cells, cells resembling monocytoid cells, small lymphocytes, and scattered immunoblasts and centroblast-like cells. A proportion of cases are accompanied by plasmacytic differentiation. The neoplastic cells reside in the marginal zones of reactive B-cell follicles and more often may extend into the interfollicular region, as well as selectively into germinal centres, forming the so-called ‘follicular colonisation’ phenomenon. Lymphoma cells occasionally invade the epithelium, forming lymphoepithelial lesions (LELs) [23].

Primary hepatic MALT lymphoma was firstly reported in 1995 by Isaacson [24,25]. Primary hepatic MALT lymphomas are mostly discovered incidentally and lack extrahepatic manifestations. They present with typical morphology and immunophenotype. The ethology of primary liver MALT lymphoma is still unclear but appears to be associated with chronic antigenic stimulation related to infectious agents or autoimmune disorders [26,27]. The underlying liver disease, such as autoimmune or infective, can support the hypothesis that chronic inflammation can stimulate the development of MALT lymphoma as seen in other organs, such as gastric MALT lymphoma with *Helicobacter pylori* infection or thyroid MALT lymphoma with Hashimoto disease [28].

From 1995 on, primary hepatic MALT lymphoma was seldom found as a primary liver neoplasm [3,4,5,6,7,8,9,10,11,12,13,14,15,16,17,18,19,20,21]. MALT lymphomas are usually low-grade lymphomas with indolent, favourable, and localised clinical course [26]. Recurrence after treatment is unusual but can be present and is most frequently discovered in the lungs [5,8].

In the literature, primary hepatic MALT lymphoma was described in 113 cases [3,4,5,6,7,8,9,10,11,12,13,14,15,16,17,18,19,20,21]. According to the systematic review of the literature, the lesions were equally distributed between sexes. Patients were mostly asymptomatic [3,6,10]. Lesions were chiefly incidentally discovered during imaging due to other complaints, on routine imaging for chronic hepatic disease follow-up or during liver transplant evaluation [27,29,30]. Pre-existing liver disease was reported in half of the patients, mostly with HBV, HCV, and primary biliary cirrhosis [6]. Cirrhosis was present in one-third of reported cases of primary MALT lymphoma [6]. The disease was not only associated with chronic inflammation and underlying liver disease, as also seen in our case, but was also found accompanying cancer (prostatic, colon, breast), drug-related hepatitis, ascariasis, gastric MALT lymphoma, and rheumatoid arthritis [3,4,5,6,7,8,9]. On the other hand, according to the literature, in half of the patients there was no underlying liver disease present at the diagnosis of primary hepatic MALT lymphoma [6].

Accurate diagnosis of primary liver MALT lymphoma is often challenging and requires histological verification either by needle biopsy or liver resection [6,7]. According to the literature, half of patients are diagnosed preoperatively with needle biopsy [6]. The main challenge is the distinguishment between primary hepatic MALT lymphoma and other primary liver cancers, such as hepatocellular carcinoma or intrahepatic cholangiocarcinoma. This is extremely difficult, especially in cases of concomitant liver disease which was also experienced in this case. According to the literature, primary MALT lymphoma was misdiagnosed mainly as hepatocellular carcinoma (in nearly 20%), intrahepatic cholangiocarcinoma, hepatic haemangioma, hepatic metastasis, pseudolymphoma, or hepatic adenoma [10].

Imaging features of primary liver MALT lymphoma on MRI are often nonspecific. On T1-weighted images, the lesion usually appears hypointense relative to the liver parenchyma, while on T2-weighted images, it is typically moderately hyperintense. Post-contrast dynamic imaging may demonstrate mild enhancement during the arterial phase, with variable enhancement patterns in the portal venous and delayed phases. Diffusion-weighted imaging often reveals restricted diffusion. Morphologically, hepatic MALT lymphoma may present as either a well-circumscribed mass or an ill-defined infiltrative lesion and can mimic other malignant tumours, as well as inflammatory or infectious processes, due to its subtle imaging features. These lymphomas may involve the portal venous system and frequently coexist with inflammation, complicating image interpretation—especially in the setting of underlying liver disease, such as primary sclerosing cholangitis, as in our case [31,32].

In this case, the imaging findings initially raised strong suspicion for intrahepatic cholangiocarcinoma, particularly in the setting of underlying PSC. The large, infiltrative-appearing, ill-defined hypovascular mass—demonstrating moderate T2 hyperintensity, marked diffusion restriction, vascular encasement or occlusion, and apparent extrahepatic extension—was characteristic of an aggressive neoplasm, with cholangiocarcinoma being the leading diagnosis in the context of PSC. However, retrospective review and histopathological confirmation revealed the lesion to be a primary liver MALT lymphoma. The imaging appearance mimicked an aggressive malignancy due to its mass-forming nature and compressive effects on vascular structures, rather than true invasion. No histological evidence of extrahepatic spread was identified, and the vascular occlusion was ultimately attributed to compression rather than infiltration [31,32].

Imaging modality that can be helpful in the preoperative diagnosis of primary MALT lymphoma, according to few reports, is CEUS [10,33,34,35]. It has been shown that CEUS can be useful in differential diagnosis of rare liver tumours [34]. There have only been six reports analysing and discussing the benefits of CEUS in settings of primary hepatic MALT lymphoma [10,33,34,35]. One of the useful characteristics of CEUS could be the uneven enhancement of the lesion in the arterial phase and uneven regression in the portal phase [35]. Moreover, CEUS can evaluate intratumoural hemodynamics in real time and allows the visualisation of penetrating blood vessels [4,10]. As some reports show, vessel penetration signs can be observed in malignant hepatic lymphomas [4,10]. As these findings are not specific for primary hepatic MALT lymphoma, histological assessment is still critical in diagnosis confirmation [4]

As discussed, lesions can be solitary, and they can show arterial phase enhancement, restricted diffusion, and vessel penetration signs [5,7,31,32]. Nevertheless, the lesions can be lacking any specific findings of primary hepatic MALT lymphoma, as was the case in our patient [5,7]. As in our case, mainly due to underlying liver disease, the condition is frequently misdiagnosed as other, more common hepatic lesions of malignant or benign potential [3,4,5,6,7,8,9,10,11,12,13,14,15,16,17,18,19,20,21]. As a result, according to the literature, these tumours are resected in up to 70% of patients [5,6,7]. In the majority of cases, resection was indicated due to the suspicion of hepatocellular carcinoma [8,10].

Due to its rarity and challenging diagnostic workup, there are still no firm guidelines for the treatment of primary hepatic MALT lymphoma. Current treatment recommendations include surgery, chemotherapy, radiotherapy, and comprehensive treatment [10]. According to the literature, around 50% of patients underwent surgical resection that was accompanied by chemotherapy [5,10]. Resection can be safely done in a minimally invasive manner with adequate oncologic outcome [9]. The recurrence of the disease is rare but can happen; therefore, regular follow-up is important [10].

Up till now, four patients with primary MALT lymphoma underwent successful LT [3,5,27]. All patients had underlying end-stage liver cirrhosis with one or multiple liver lesions believed to represent hepatocellular carcinoma secondary to liver cirrhosis. Therefore, LT was performed [5]. In our case, the tumour was successfully resected with clear margins with extended right hepatectomy. As the patient had an underlying liver disease but no cirrhosis jet, the volume of the liver remnant was not sufficient for its adequate functioning. Therefore, the patient developed small-for-size syndrome, leading to liver failure requiring LT. If the patient had no prior liver disease, the volume of the remnant would probably be sufficient for normal liver function, and no further treatment would be necessary.

This case highlights the diagnostic challenges of differentiating primary hepatic MALT lymphoma from cholangiocarcinoma on imaging, especially in patients with underlying PSC. Preoperative confirmation of the disease could potentially change treatment course in our patient. Therefore, a serious surgical complication with development of small-for-size syndrome after the major hepatectomy could potentially be prevented. If lymphoma would be confirmed preoperatively, chemotherapy treatment would be offered to the patient, regarding the extent of the disease. If the liver function would worsen during the chemotherapy due to therapy or underlying PSC, the patient would be a candidate for LT.

## 4. Conclusions

Primary hepatic MALT lymphoma is an extremely rare liver lesion but should be kept in mind in differential diagnosis of liver lesions in patients with chronic viral infection or autoimmune disease, especially in settings of cirrhosis. Moreover, a high level of suspicion must be raised in young patients with solitary liver mass and autoimmune liver disease. Although rare, hepatic MALT lymphoma should be considered in the differential diagnosis of liver masses with atypical features or when histology and imaging findings are discordant. Awareness of this potential mimic can prevent misdiagnosis and guide appropriate management. As there are no clear guidelines for the treatment of primary MALT lymphoma, one must tailor treatment according to the patient. In most cases, surgical resection is the best way to achieve elimination of the disease. However, with most of the patients presenting with the underlying liver disease, sometimes radical resection with preserved liver function of the remnant is not possible; therefore, these patients may benefit from liver transplantation in cases of chemotherapy failure or development of liver failure due to chemotherapy, lymphoma, or underlying liver disease.

## Data Availability

All available data are presented in the case.
